# Supplementary material for: Can Measurements of Inflammatory Biomarkers Be Used to Spot Respiratory Viral Infections?
Source: Viruses. 2020 Oct 17;12(10):1175. doi: 10.3390/v12101175 (PMC7594027; doi:10.3390/v12101175)
Supplement: Supplementary file 1 [file viruses-12-01175-s001.zip › SI_revised/Supplementary Figures_S1 to S20_revised.pdf]

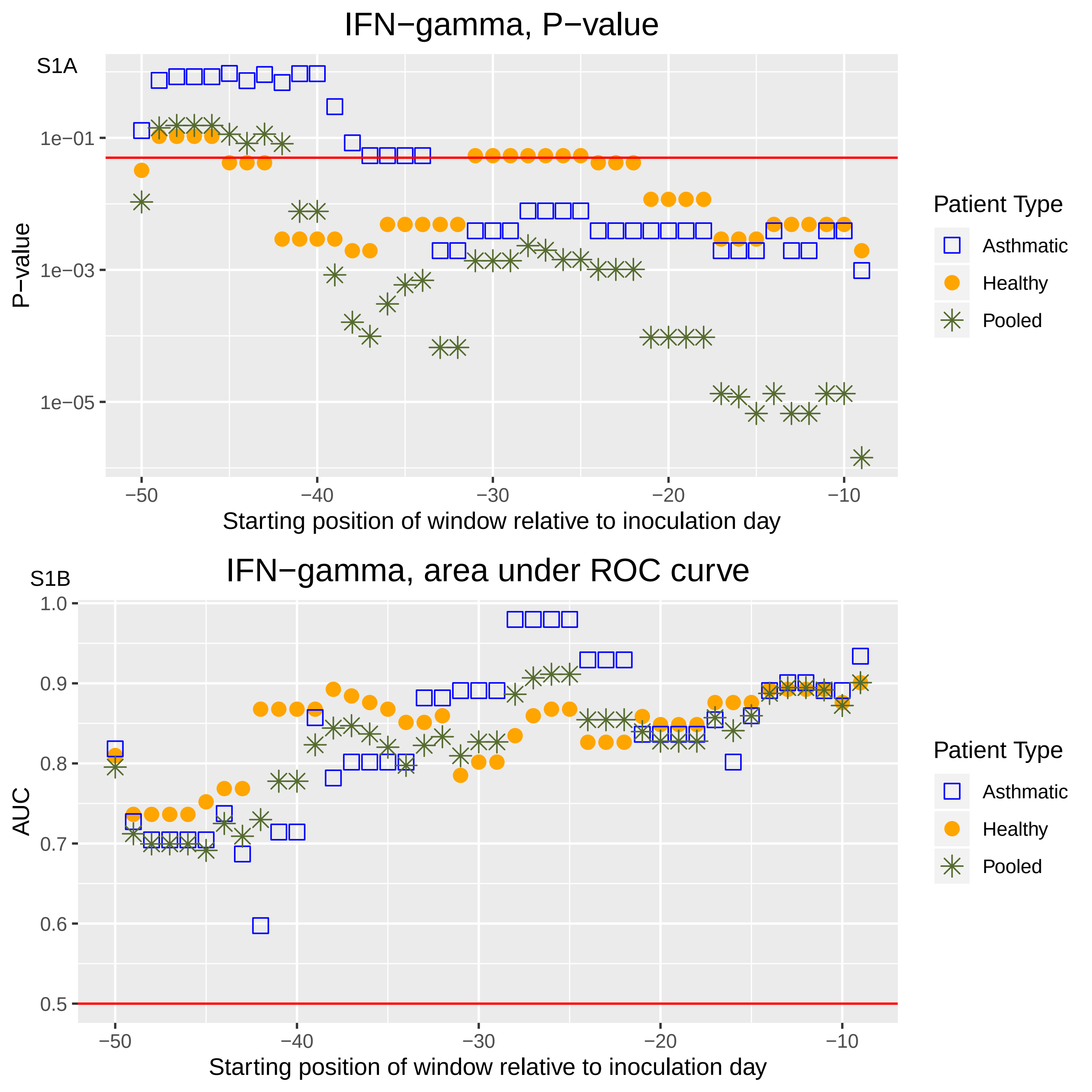

IFN−gamma, P−value
S1A
P−value
1e−01
1e−03
1e−05
−50
−40
−30
−20
−10
Starting position of window relative to inoculation day
Patient Type
Asthmatic
Healthy
Pooled
IFN−gamma, area under ROC curve
S1B
AUC
1.0
0.9
0.8
0.7
0.6
0.5
−50
−40
−30
−20
−10
Starting position of window relative to inoculation day
Patient Type
Asthmatic
Healthy
Pooled

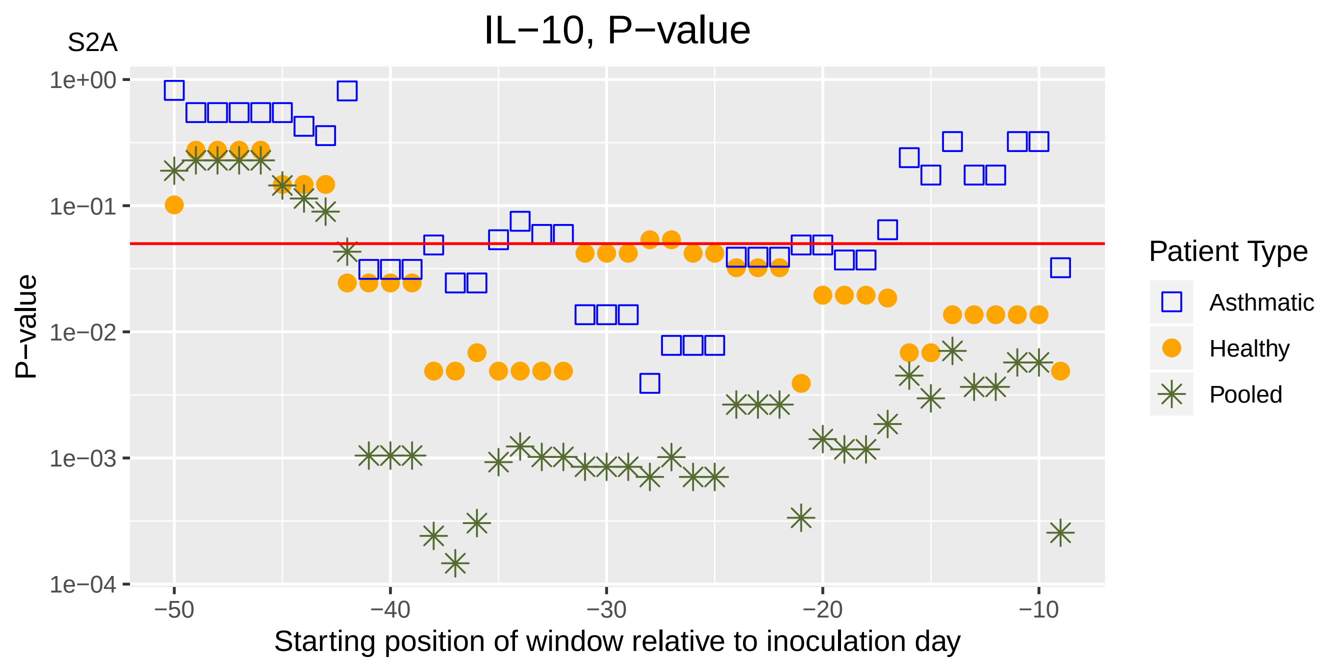
S2A
IL−10, P−value
P−value
1e+00
1e−01
1e−02
1e−03
1e−04
−50
−40
−30
−20
−10
Starting position of window relative to inoculation day
Patient Type
Asthmatic
Healthy
Pooled

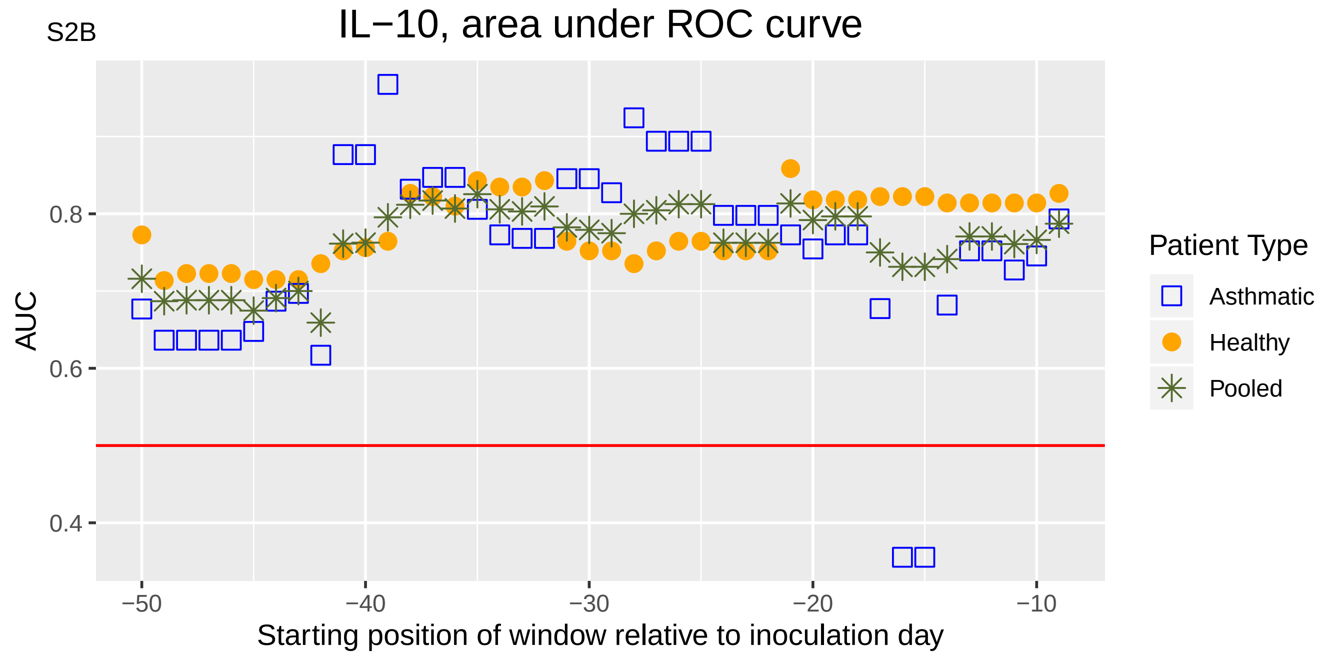
S2B
IL−10, area under ROC curve
AUC
0.8
0.6
0.4
−50
−40
−30
−20
−10
Starting position of window relative to inoculation day
Patient Type
Asthmatic
Healthy
Pooled

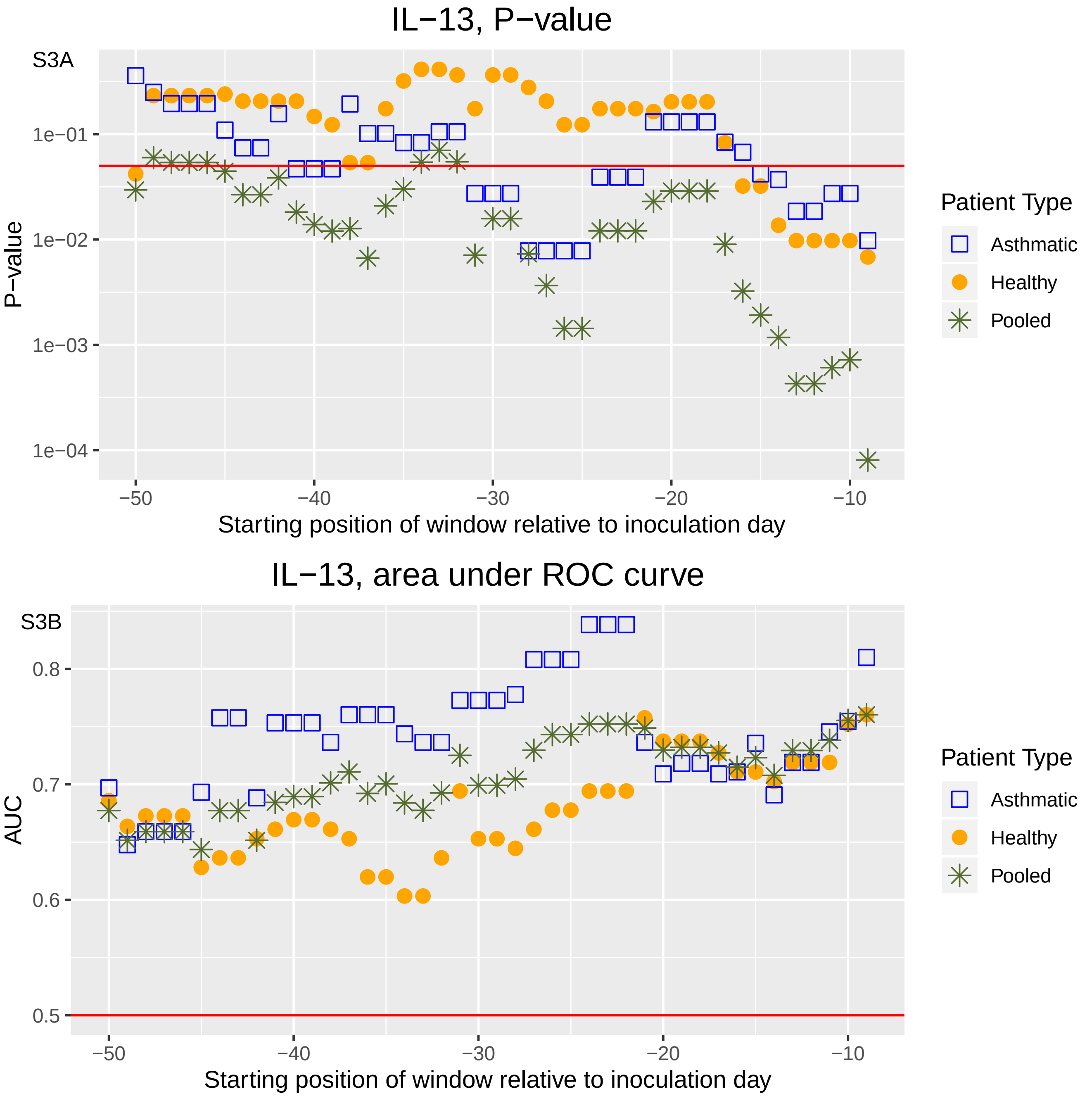
IL−13, P−value
S3A
P−value
1e−01
1e−02
1e−03
1e−04
−50
−40
−30
−20
−10
Starting position of window relative to inoculation day
Patient Type
Asthmatic
Healthy
Pooled
IL−13, area under ROC curve
S3B
AUC
0.8
0.7
0.6
0.5
−50
−40
−30
−20
−10
Starting position of window relative to inoculation day
Patient Type
Asthmatic
Healthy
Pooled

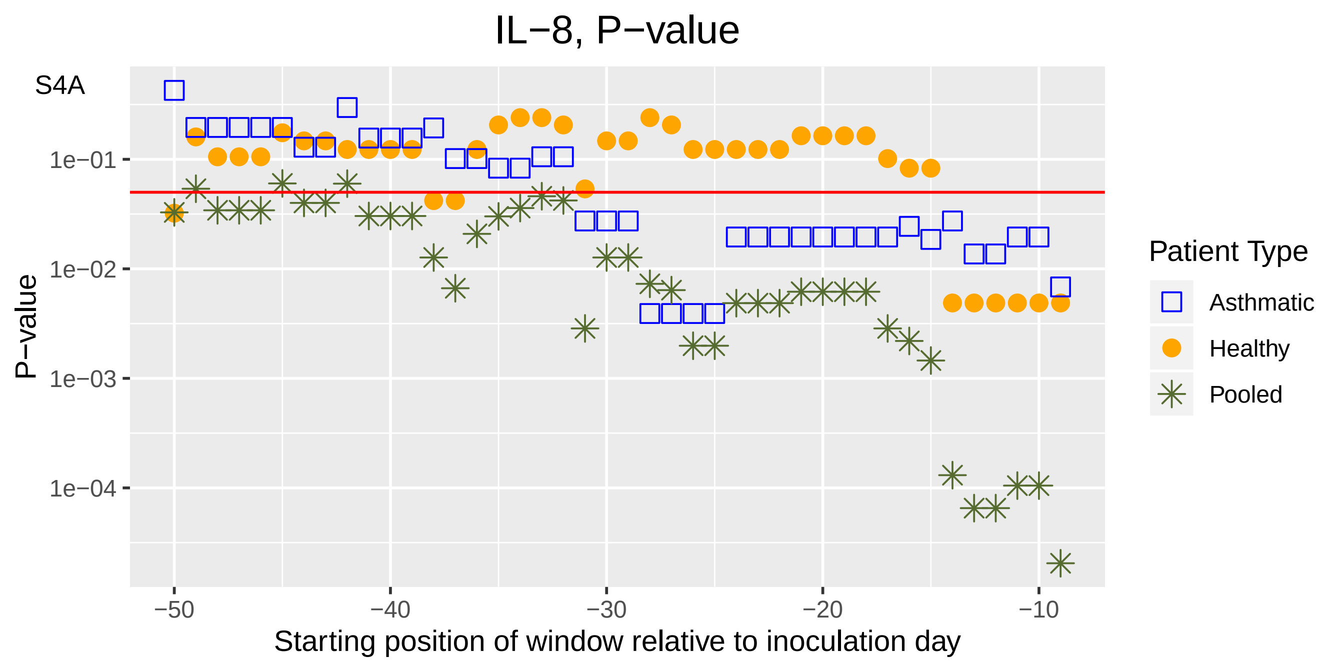
IL−8, P−value
S4A
P−value
1e−01
1e−02
1e−03
1e−04
−50
−40
−30
−20
−10
Starting position of window relative to inoculation day
Patient Type
Asthmatic
Healthy
Pooled

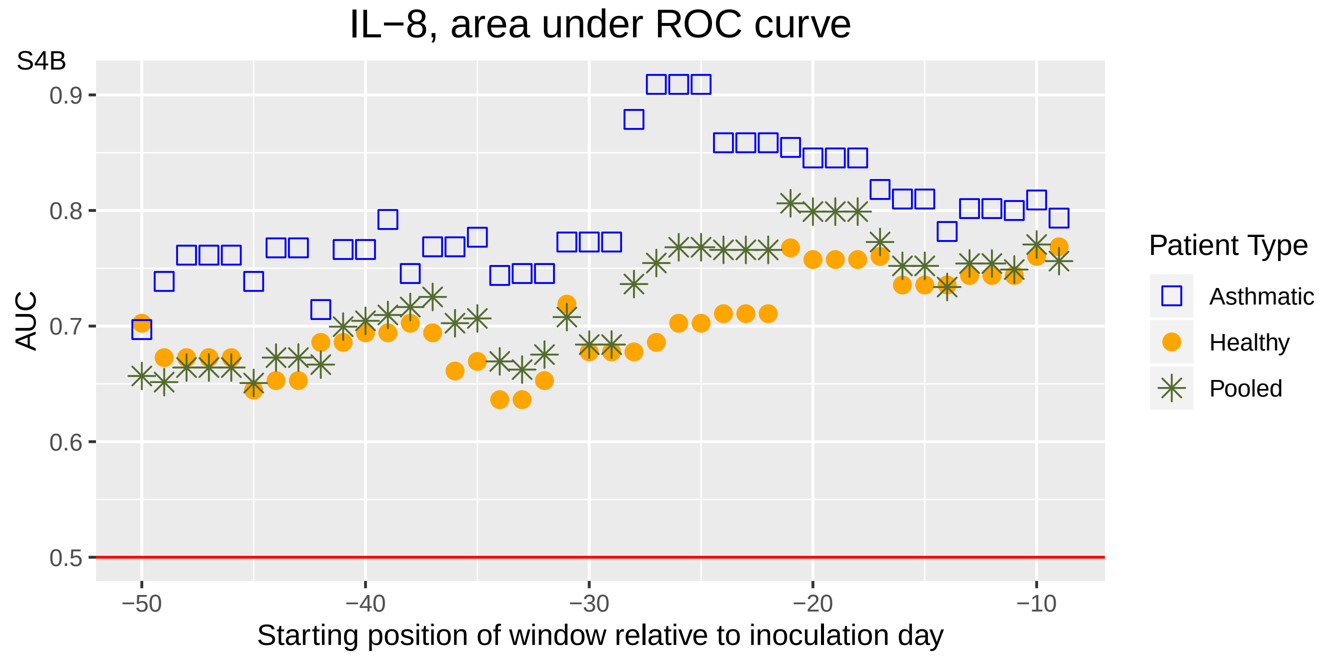
IL−8, area under ROC curve
S4B
AUC
0.9
0.8
0.7
0.6
0.5
−50
−40
−30
−20
−10
Starting position of window relative to inoculation day
Patient Type
Asthmatic
Healthy
Pooled

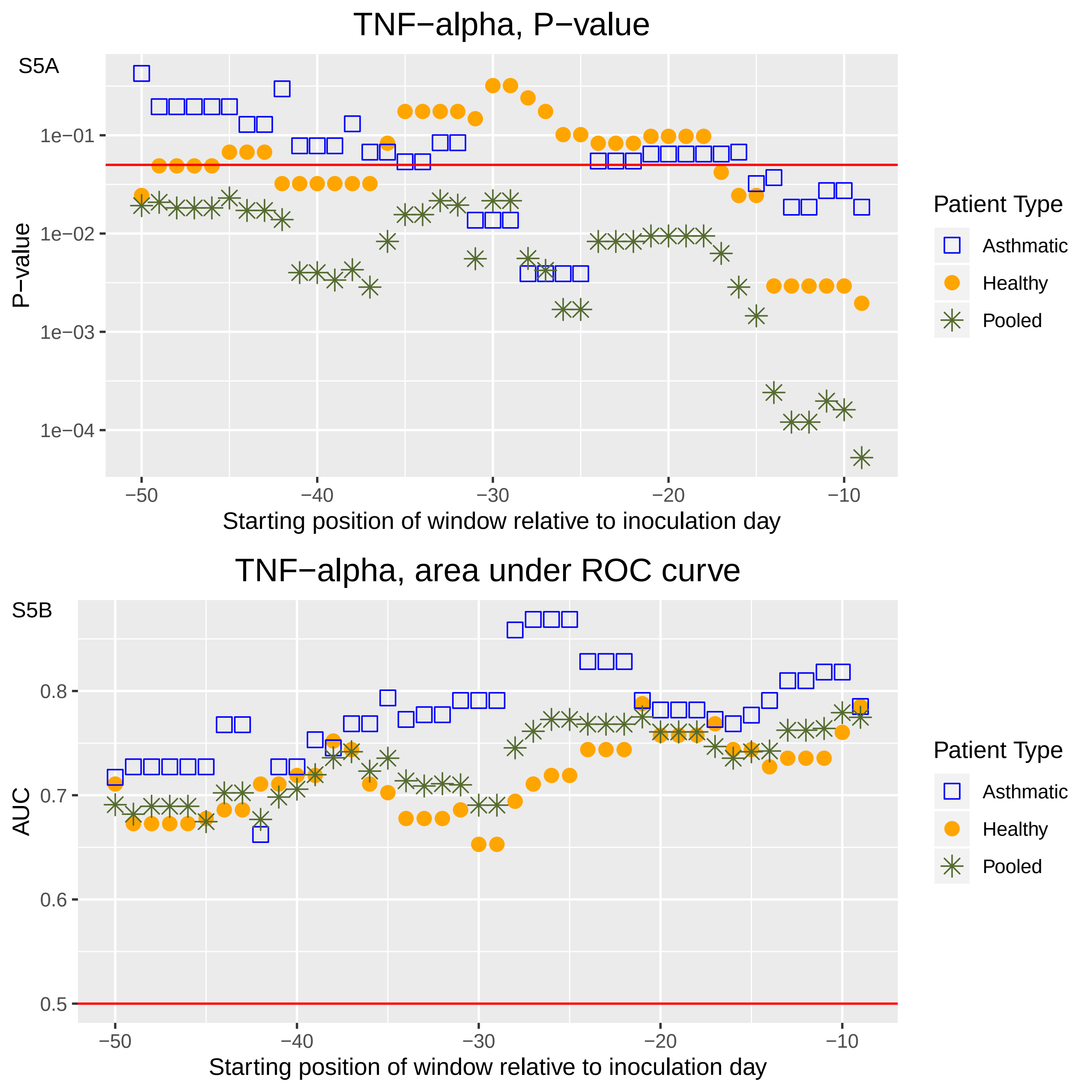
TNF−alpha, P−value
S5A
P−value
1e−01
1e−02
1e−03
1e−04
−50
−40
−30
−20
−10
Starting position of window relative to inoculation day
Patient Type
Asthmatic
Healthy
Pooled
TNF−alpha, area under ROC curve
S5B
AUC
0.8
0.7
0.6
0.5
−50
−40
−30
−20
−10
Starting position of window relative to inoculation day
Patient Type
Asthmatic
Healthy
Pooled

S6A

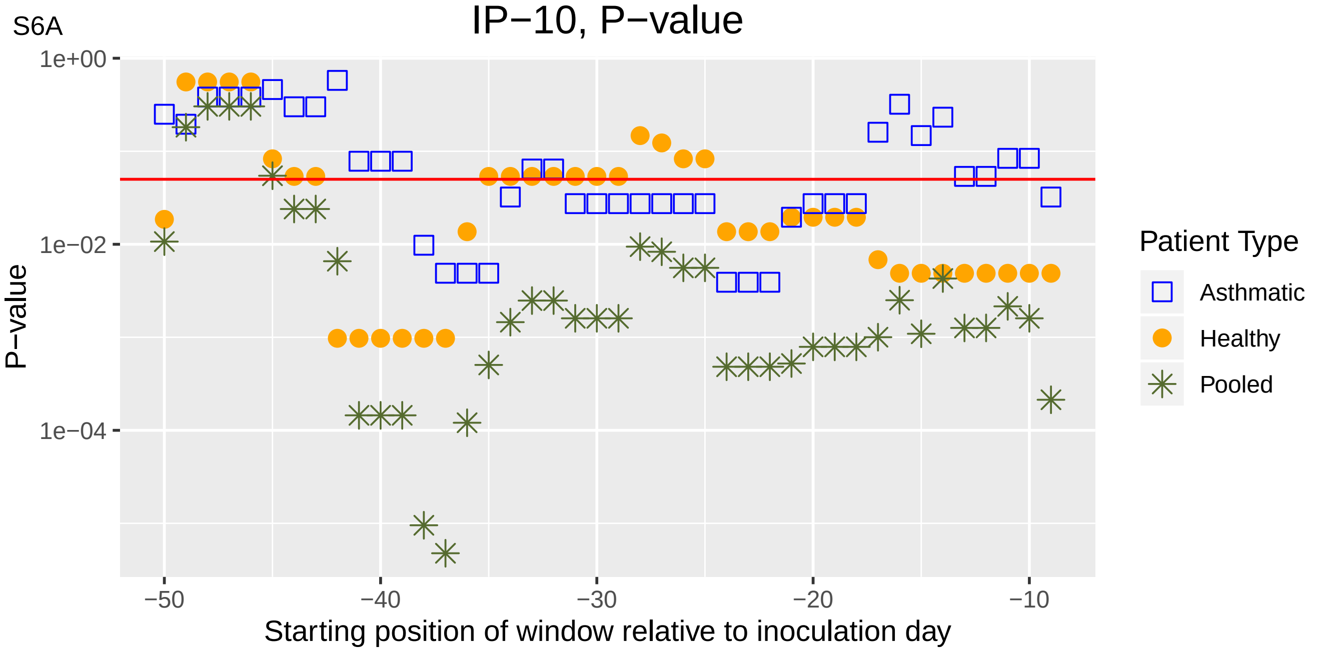
IP−10, P−value
1e+00
1e−02
1e−04
P−value
−50
−40
−30
−20
−10
Starting position of window relative to inoculation day
Patient Type
Asthmatic
Healthy
Pooled

S6B

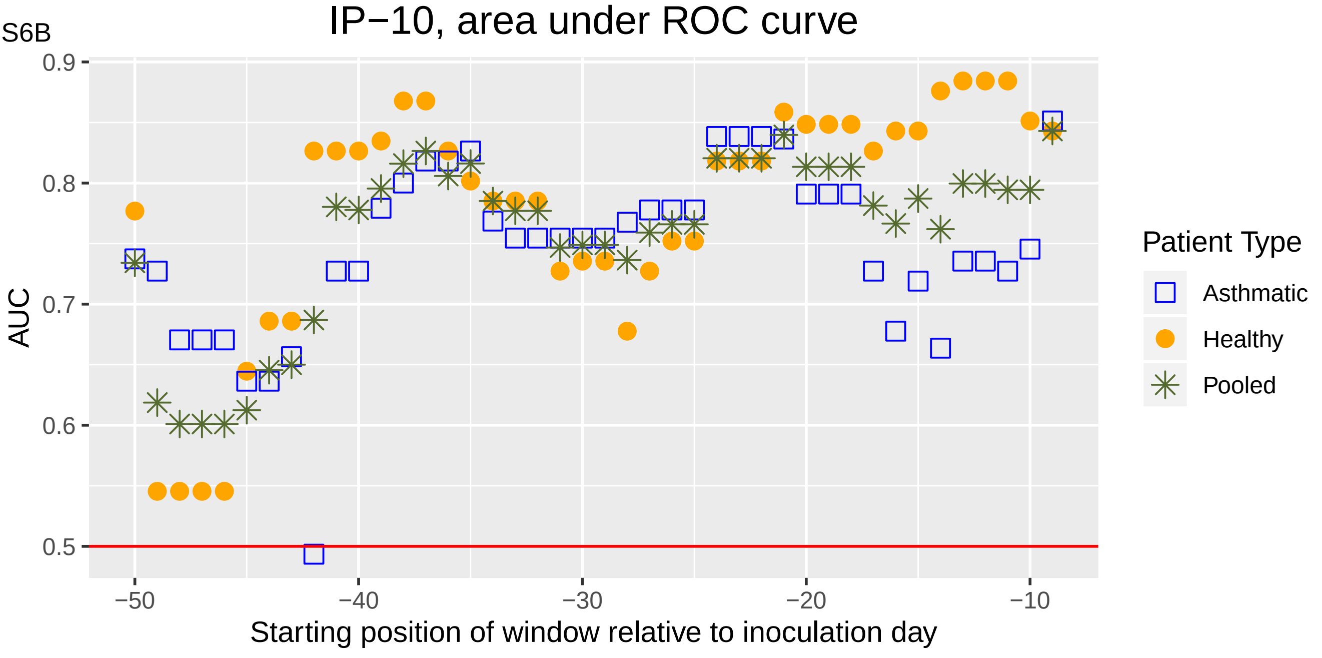
IP−10, area under ROC curve
0.9
0.8
0.7
0.6
0.5
AUC
−50
−40
−30
−20
−10
Starting position of window relative to inoculation day
Patient Type
Asthmatic
Healthy
Pooled

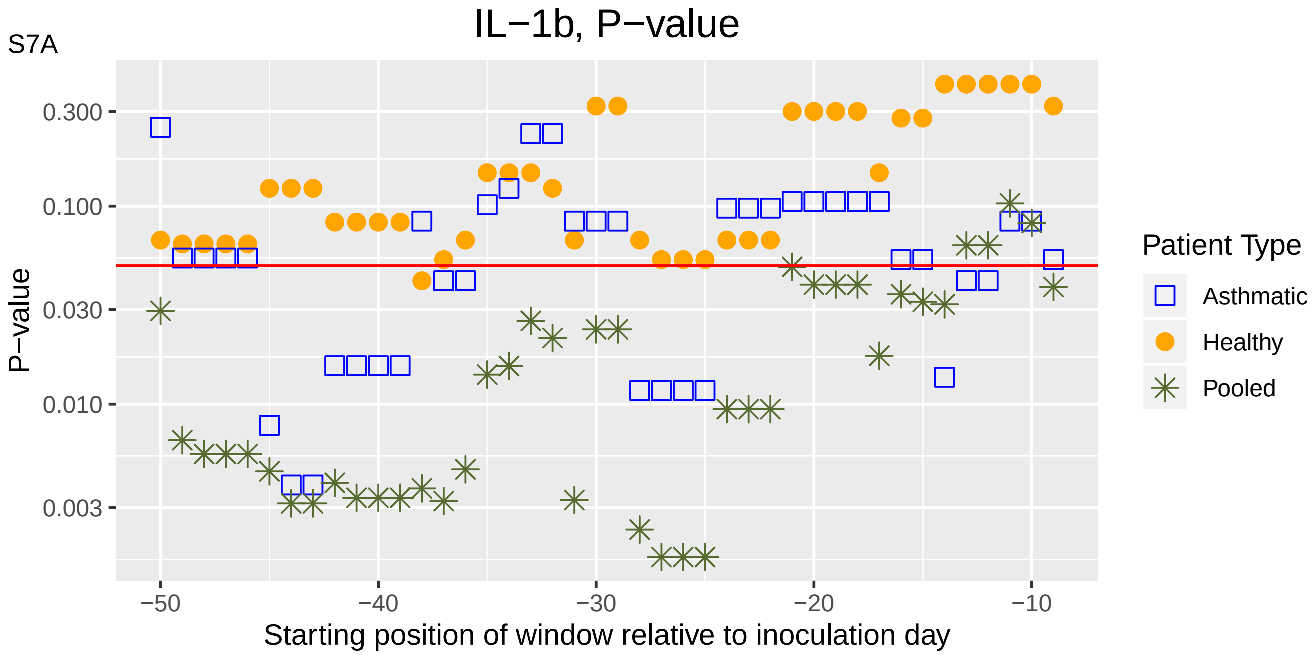

S7A
IL−1b, P−value
P−value
0.300
0.100
0.030
0.010
0.003
−50
−40
−30
−20
−10
Starting position of window relative to inoculation day
Patient Type
Asthmatic
Healthy
Pooled

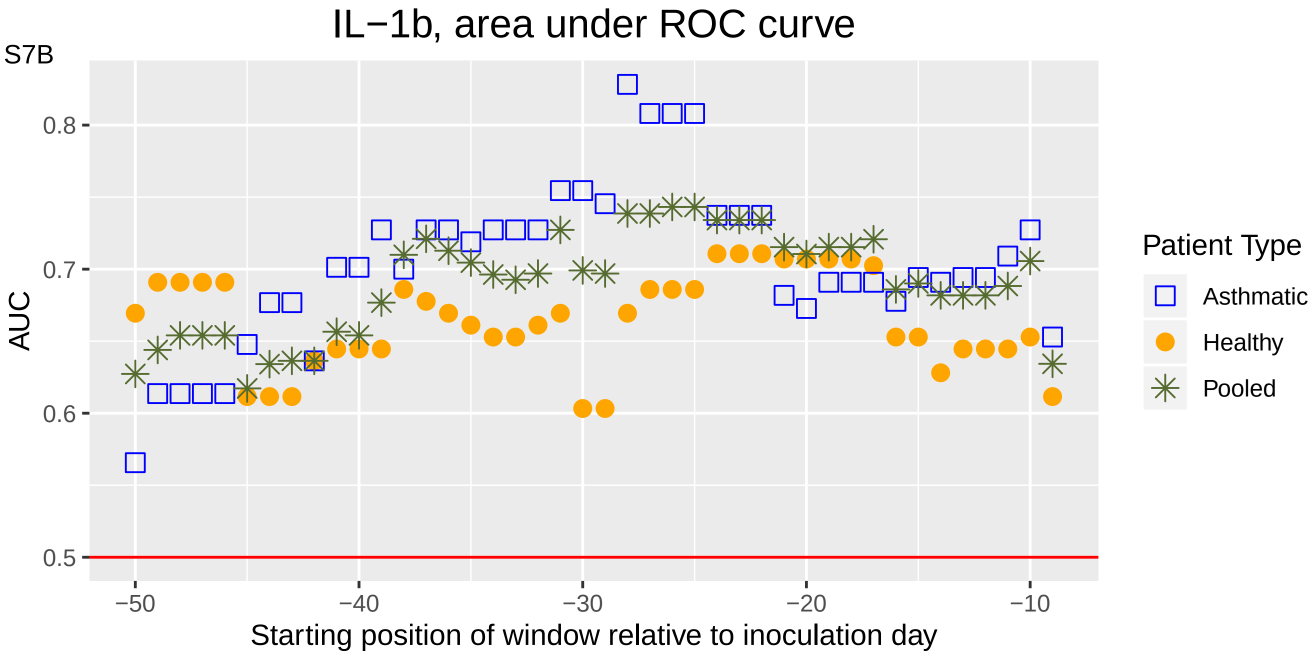

S7B
IL−1b, area under ROC curve
AUC
0.8
0.7
0.6
0.5
−50
−40
−30
−20
−10
Starting position of window relative to inoculation day
Patient Type
Asthmatic
Healthy
Pooled

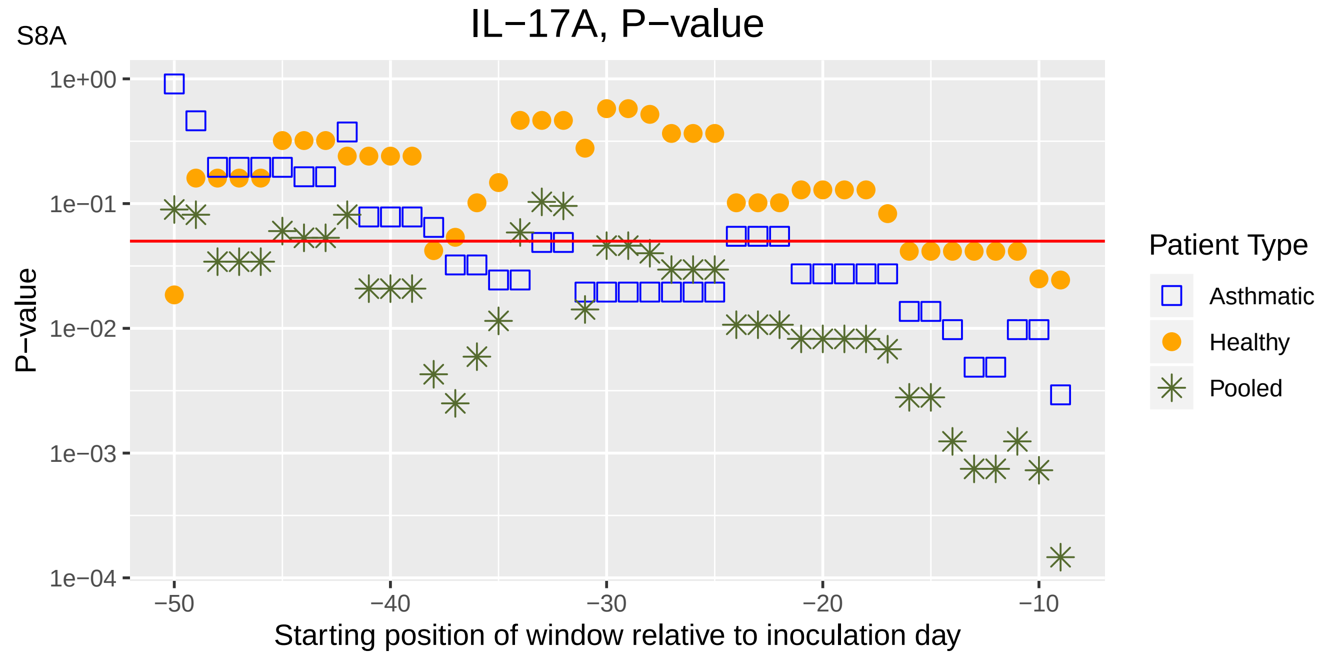
S8A
IL−17A, P−value
P−value
1e+00
1e−01
1e−02
1e−03
1e−04
−50
−40
−30
−20
−10
Starting position of window relative to inoculation day
Patient Type
Asthmatic
Healthy
Pooled

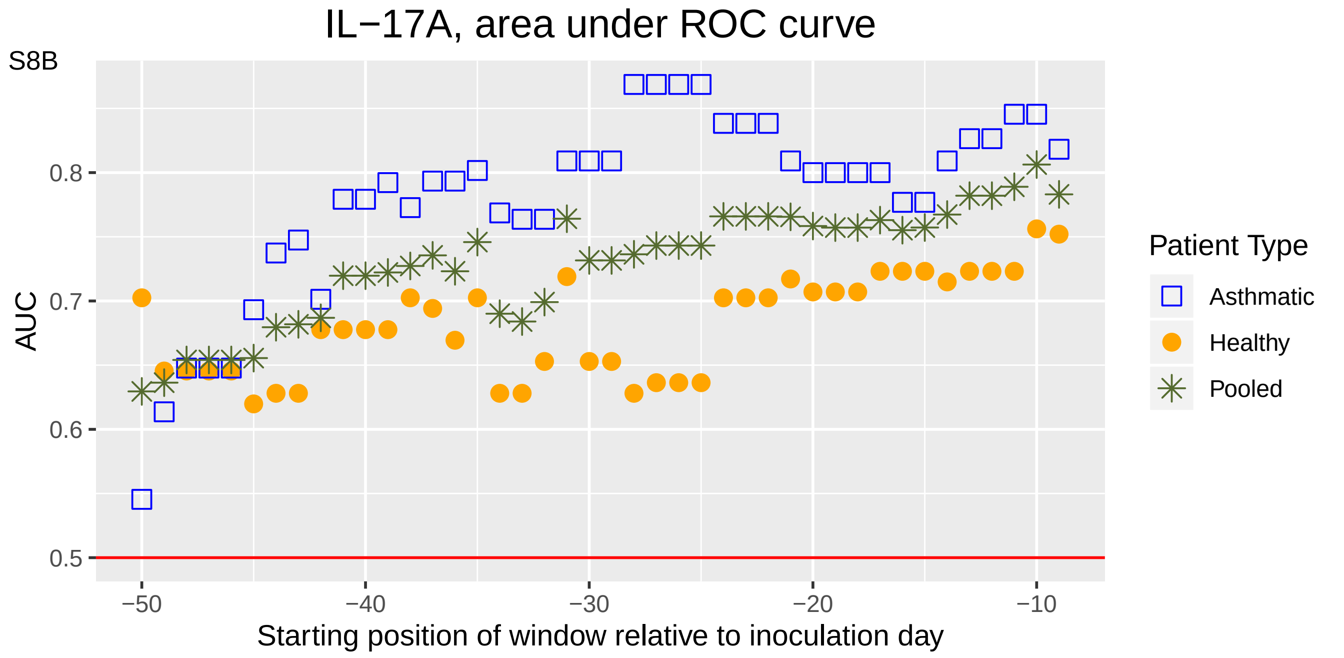
IL−17A, area under ROC curve
S8B
AUC
0.8
0.7
0.6
0.5
−50
−40
−30
−20
−10
Starting position of window relative to inoculation day
Patient Type
Asthmatic
Healthy
Pooled

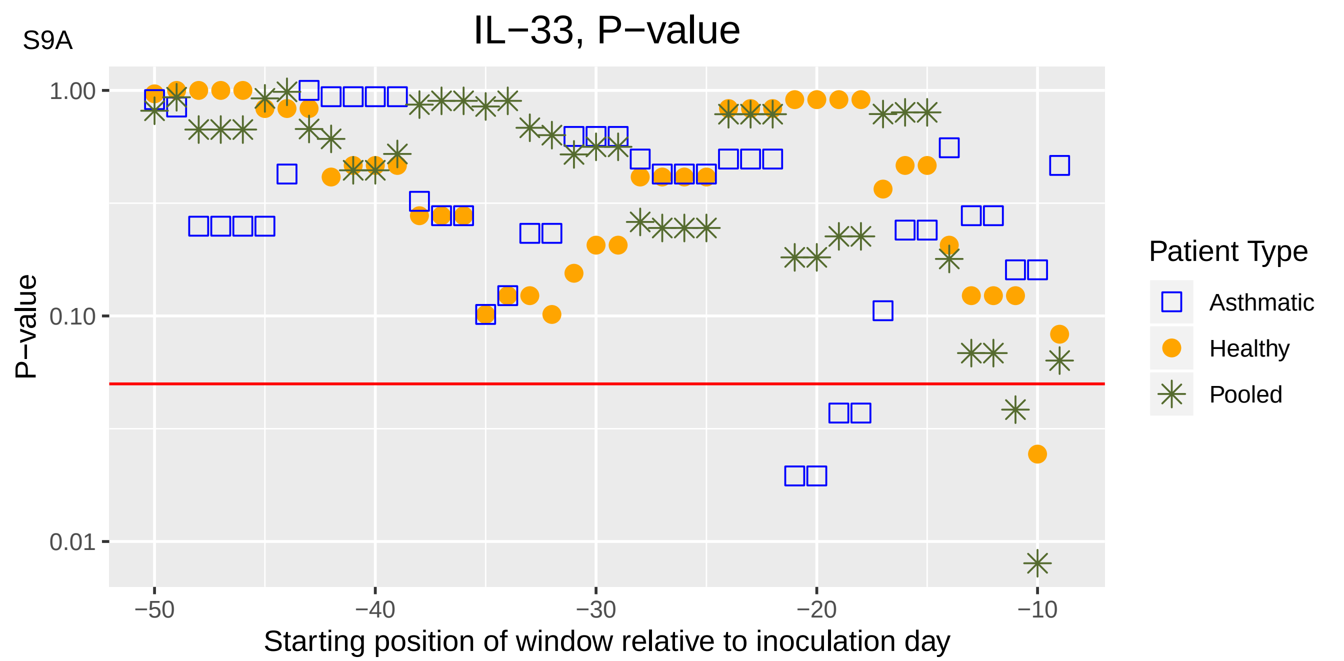
S9A
IL−33, P−value
P−value
1.00
0.10
0.01
−50
−40
−30
−20
−10
Starting position of window relative to inoculation day
Patient Type
Asthmatic
Healthy
Pooled

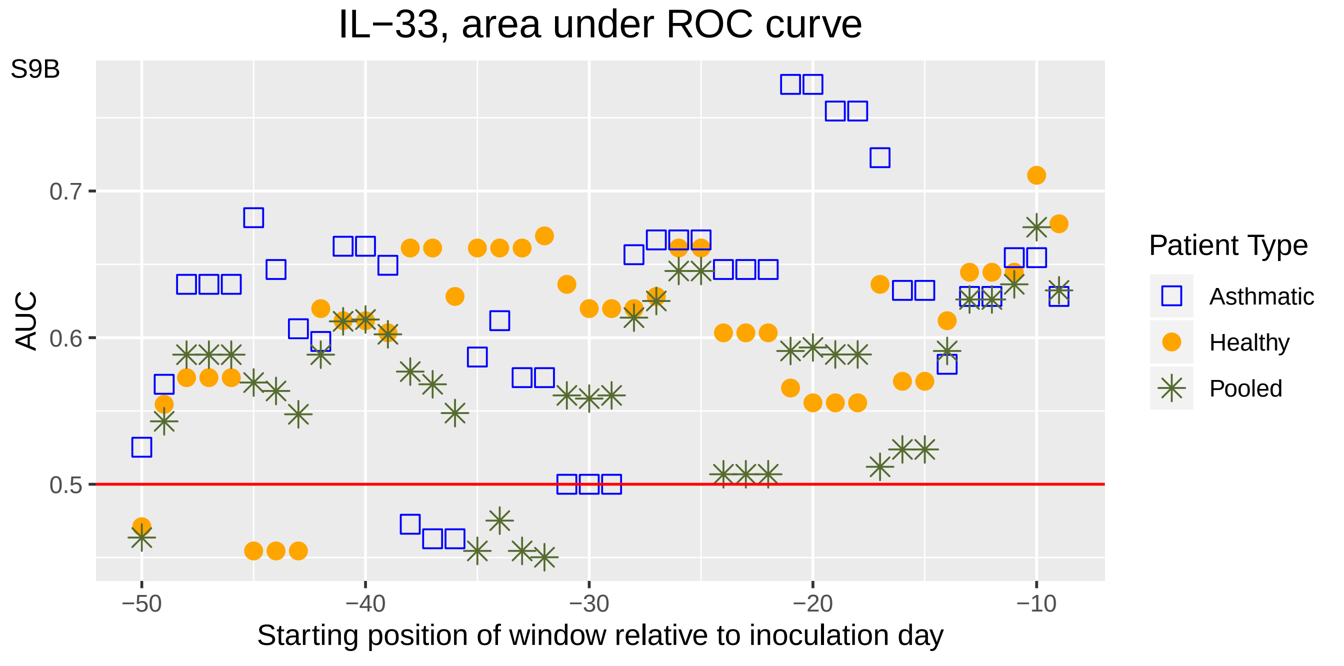
IL−33, area under ROC curve
S9B
AUC
0.7
0.6
0.5
−50
−40
−30
−20
−10
Starting position of window relative to inoculation day
Patient Type
Asthmatic
Healthy
Pooled

S10A

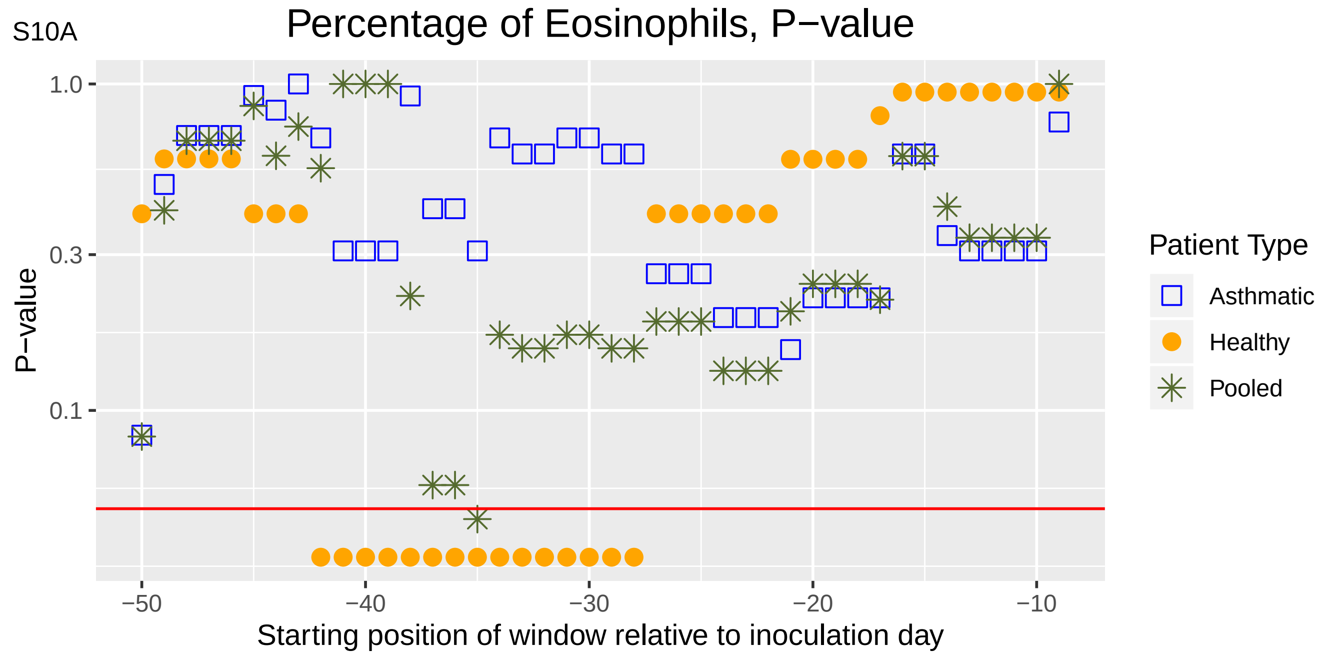
Percentage of Eosinophils, P−value
P−value
1.0
0.3
0.1
−50
−40
−30
−20
−10
Starting position of window relative to inoculation day
Patient Type
Asthmatic
Healthy
Pooled

S10B

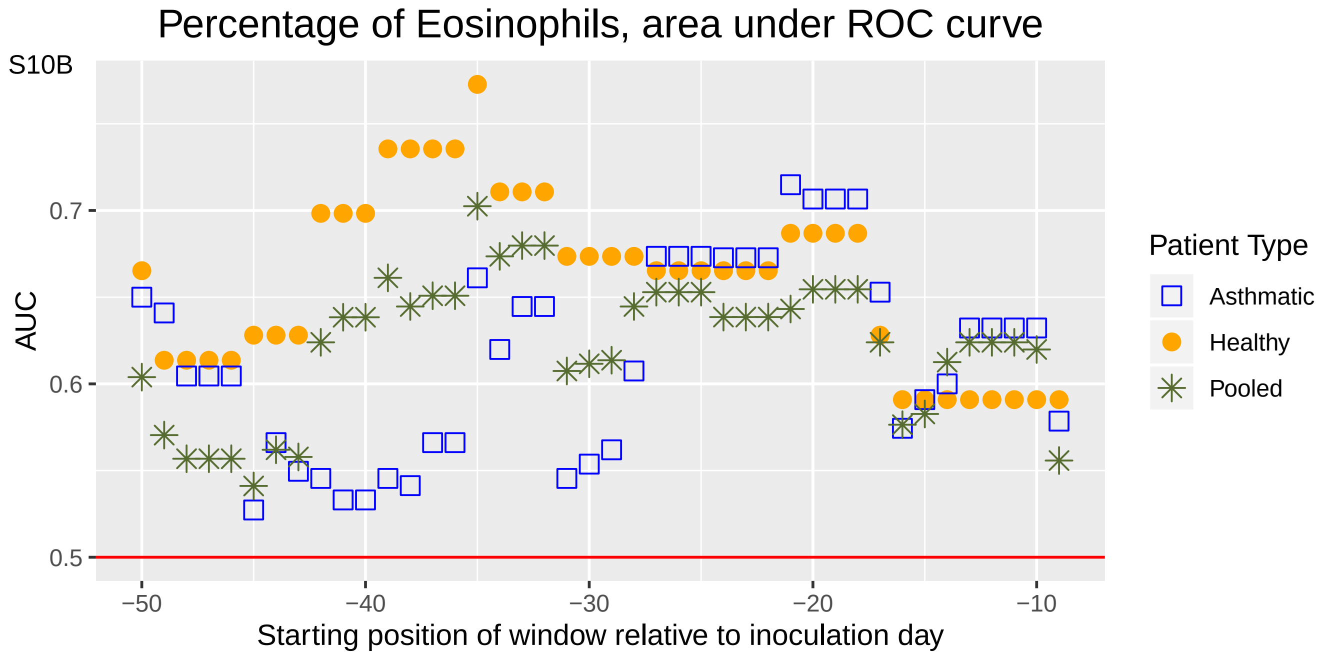
Percentage of Eosinophils, area under ROC curve
AUC
0.7
0.6
0.5
−50
−40
−30
−20
−10
Starting position of window relative to inoculation day
Patient Type
Asthmatic
Healthy
Pooled

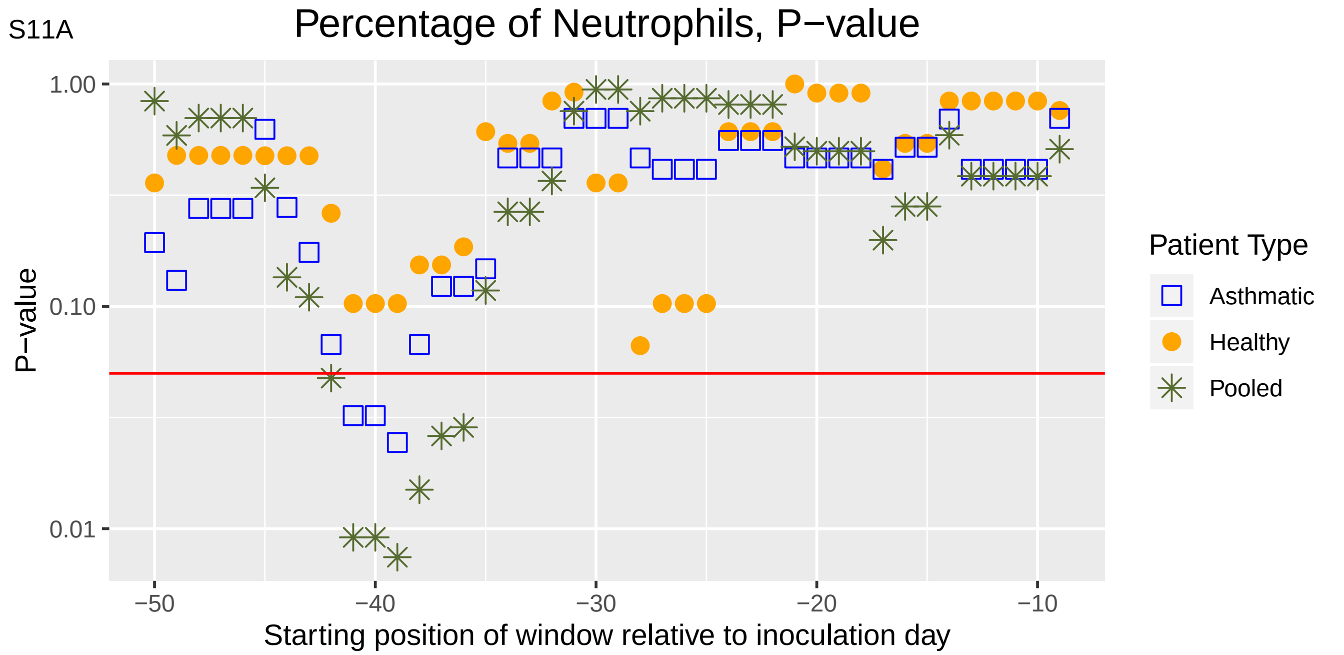
S11A
Percentage of Neutrophils, P−value
P−value
1.00
0.10
0.01
−50
−40
−30
−20
−10
Starting position of window relative to inoculation day
Patient Type
Asthmatic
Healthy
Pooled

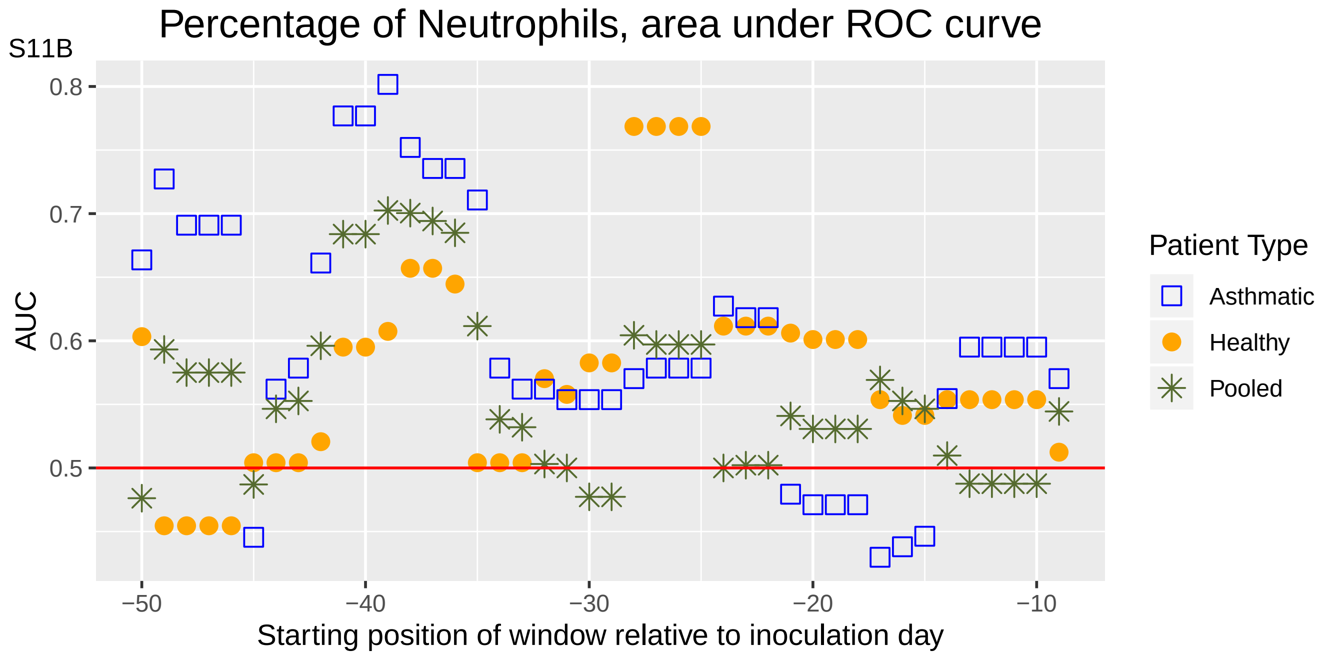
Percentage of Neutrophils, area under ROC curve
S11B
AUC
0.8
0.7
0.6
0.5
−50
−40
−30
−20
−10
Starting position of window relative to inoculation day
Patient Type
Asthmatic
Healthy
Pooled

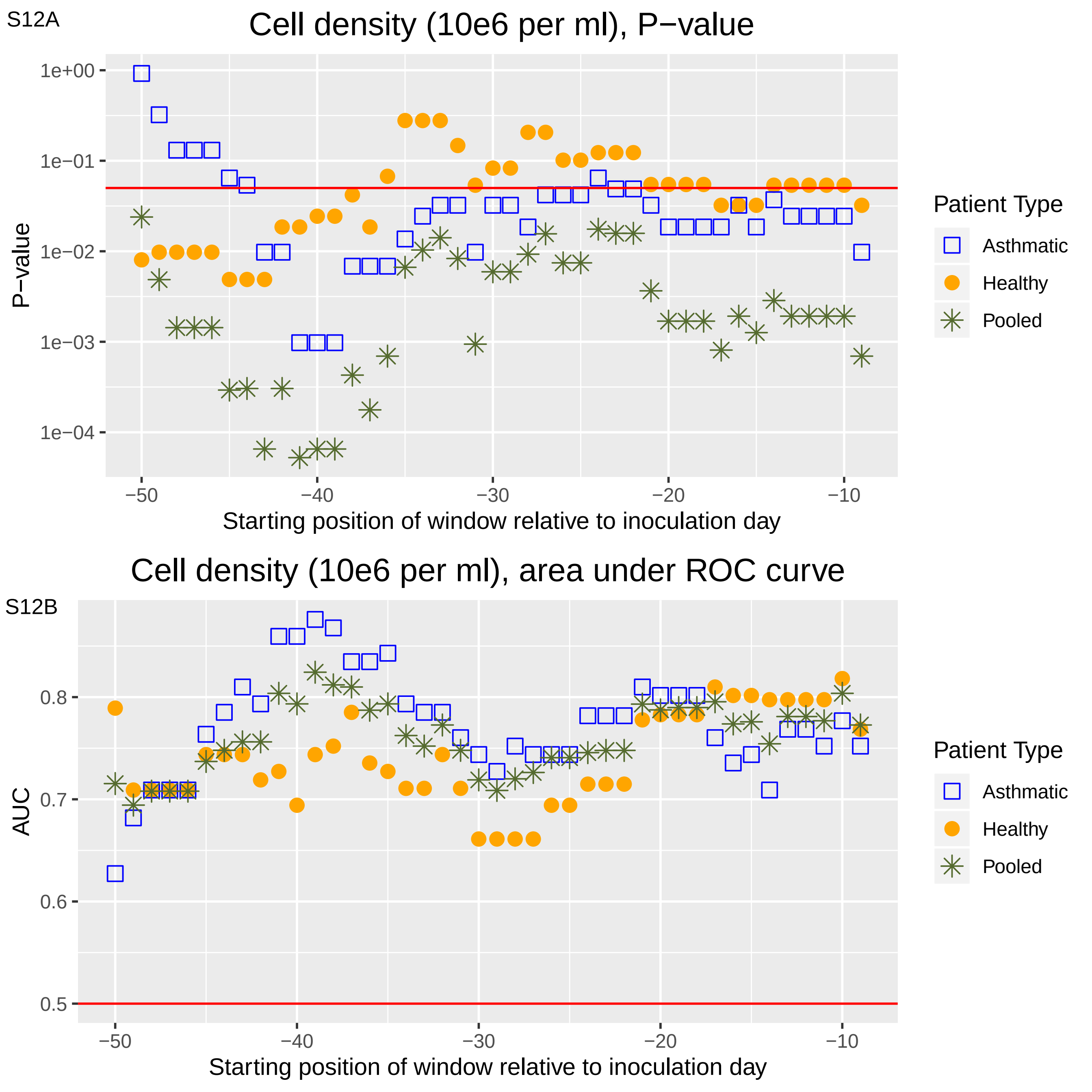

S12A
Cell density (10e6 per ml), P−value
P−value
1e+00
1e−01
1e−02
1e−03
1e−04
−50
−40
−30
−20
−10
Starting position of window relative to inoculation day
Patient Type
Asthmatic
Healthy
Pooled
Cell density (10e6 per ml), area under ROC curve
S12B
AUC
0.8
0.7
0.6
0.5
−50
−40
−30
−20
−10
Starting position of window relative to inoculation day
Patient Type
Asthmatic
Healthy
Pooled

S13A

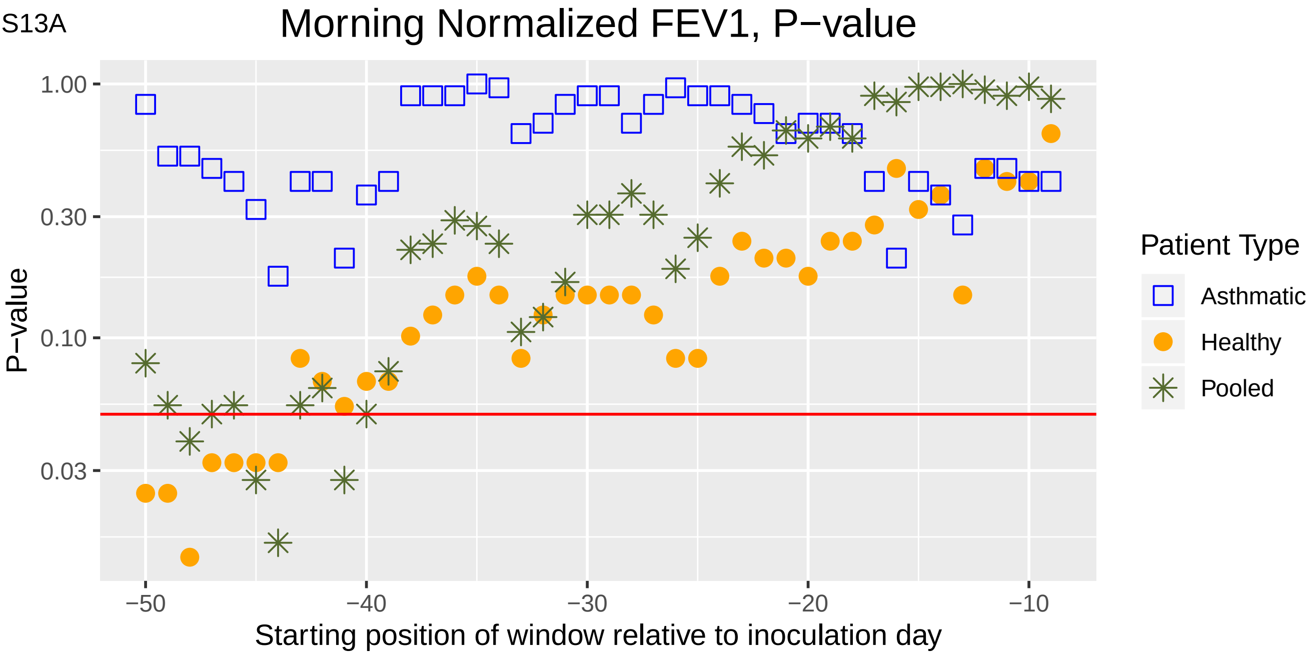
Morning Normalized FEV1, P−value
P−value
1.00
0.30
0.10
0.03
−50
−40
−30
−20
−10
Starting position of window relative to inoculation day
Patient Type
Asthmatic
Healthy
Pooled

S13B

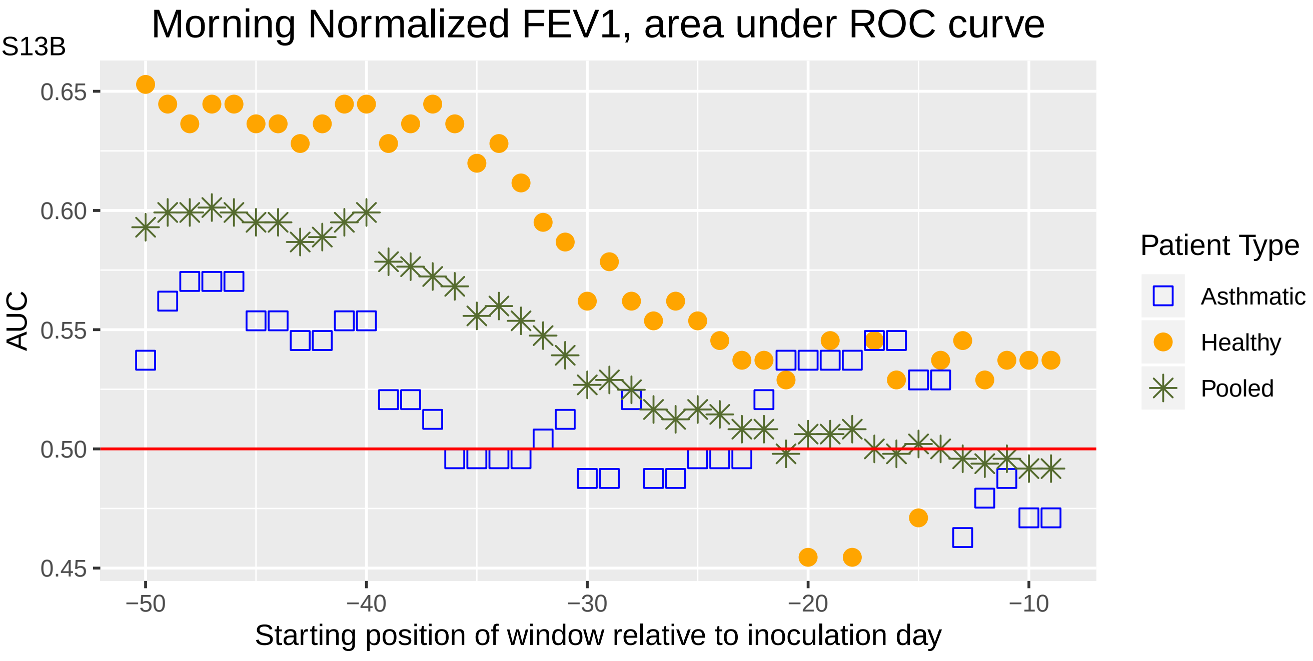
Morning Normalized FEV1, area under ROC curve
AUC
0.65
0.60
0.55
0.50
0.45
−50
−40
−30
−20
−10
Starting position of window relative to inoculation day
Patient Type
Asthmatic
Healthy
Pooled

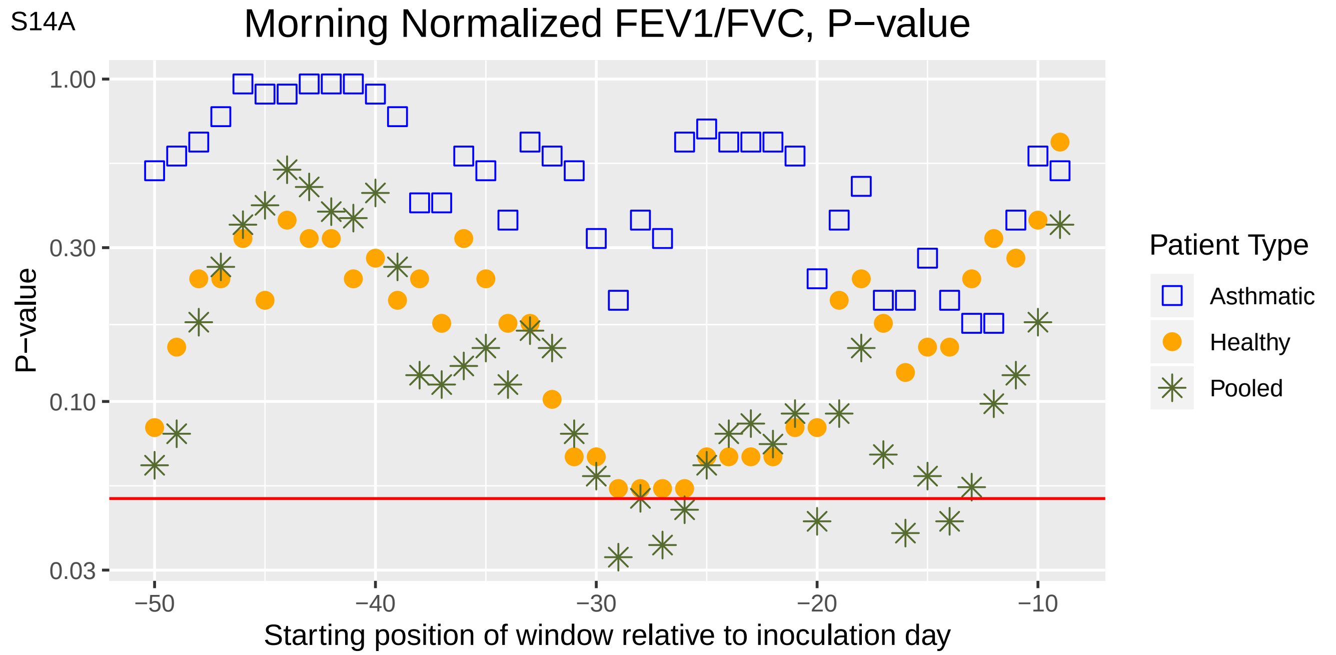
S14A
Morning Normalized FEV1/FVC, P−value
P−value
1.00
0.30
0.10
0.03
−50
−40
−30
−20
−10
Starting position of window relative to inoculation day
Patient Type
Asthmatic
Healthy
Pooled

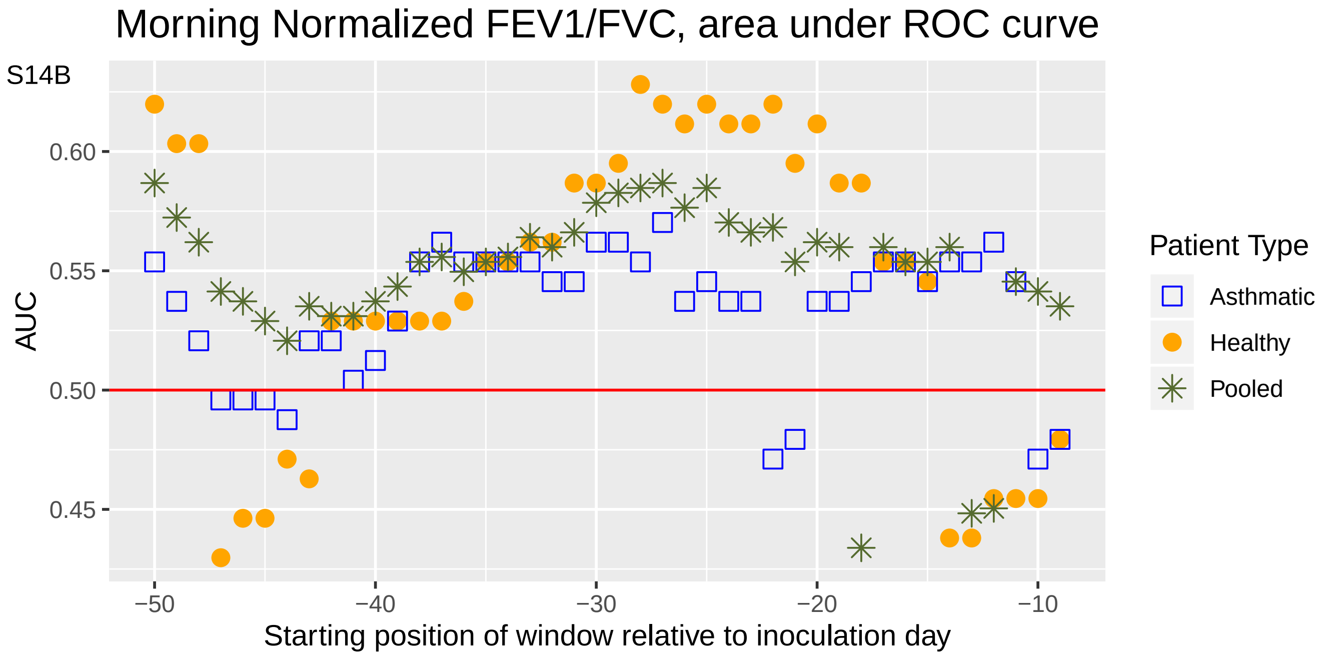
Morning Normalized FEV1/FVC, area under ROC curve
S14B
AUC
0.60
0.55
0.50
0.45
−50
−40
−30
−20
−10
Starting position of window relative to inoculation day
Patient Type
Asthmatic
Healthy
Pooled

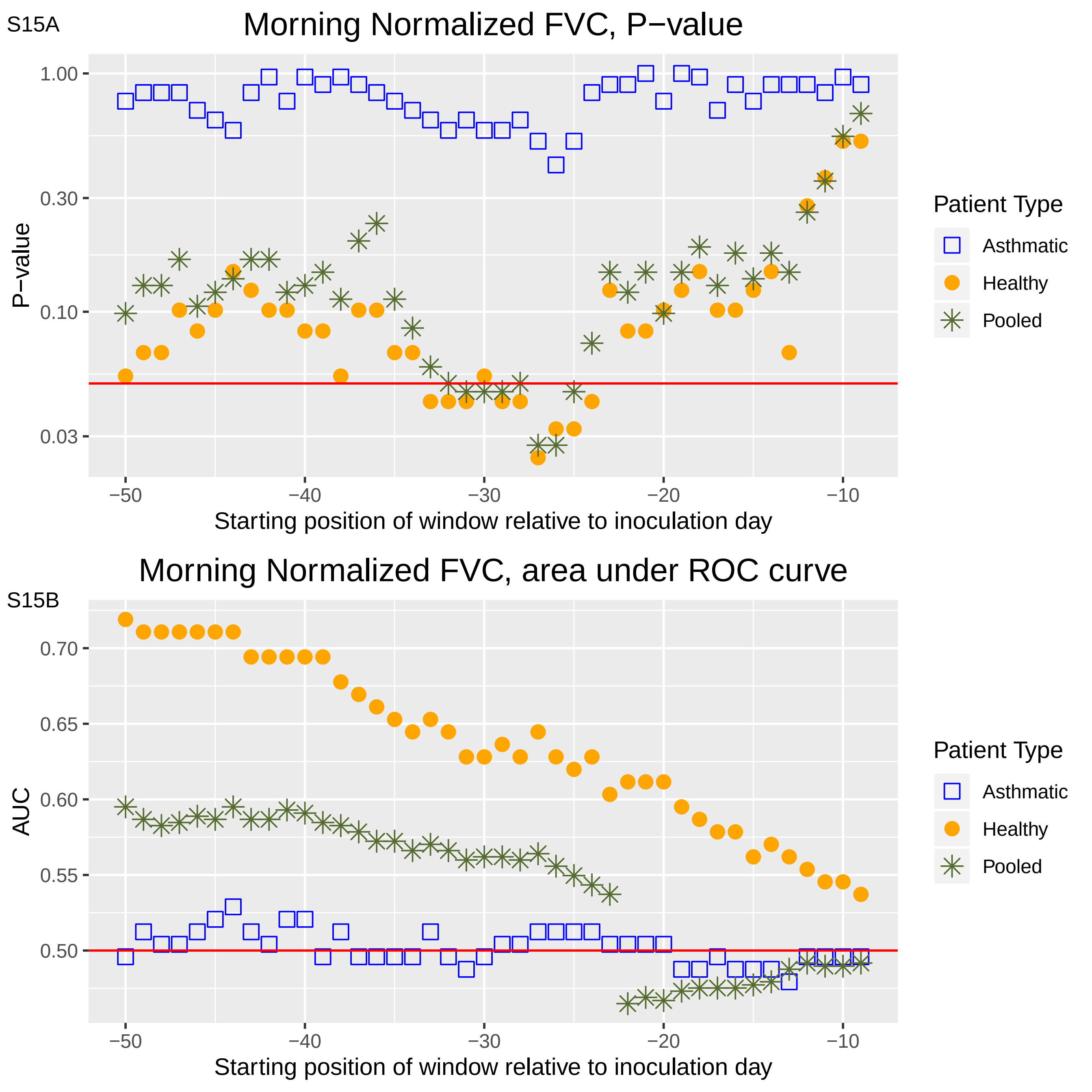
S15A
Morning Normalized FVC, P−value
P−value
1.00
0.30
0.10
0.03
−50
−40
−30
−20
−10
Starting position of window relative to inoculation day
Patient Type
Asthmatic
Healthy
Pooled
Morning Normalized FVC, area under ROC curve
S15B
AUC
0.70
0.65
0.60
0.55
0.50
−50
−40
−30
−20
−10
Starting position of window relative to inoculation day
Patient Type
Asthmatic
Healthy
Pooled

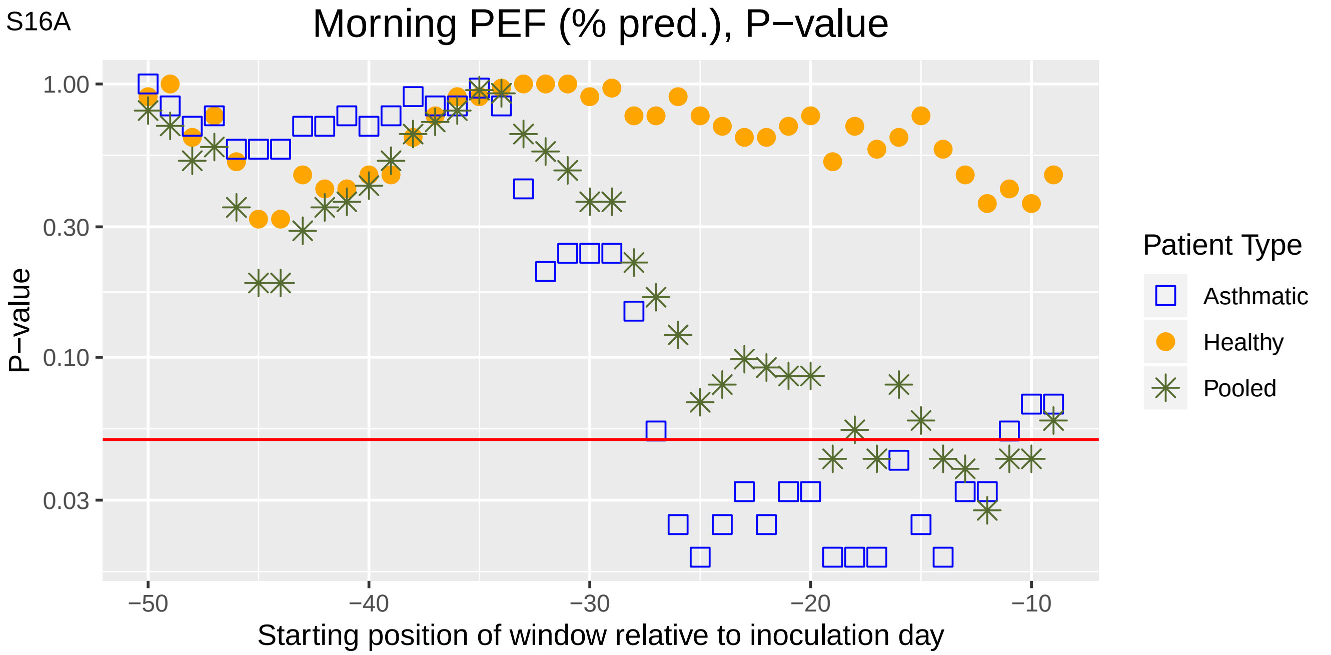
S16A
Morning PEF (% pred.), P−value
P−value
1.00
0.30
0.10
0.03
−50
−40
−30
−20
−10
Starting position of window relative to inoculation day
Patient Type
Asthmatic
Healthy
Pooled

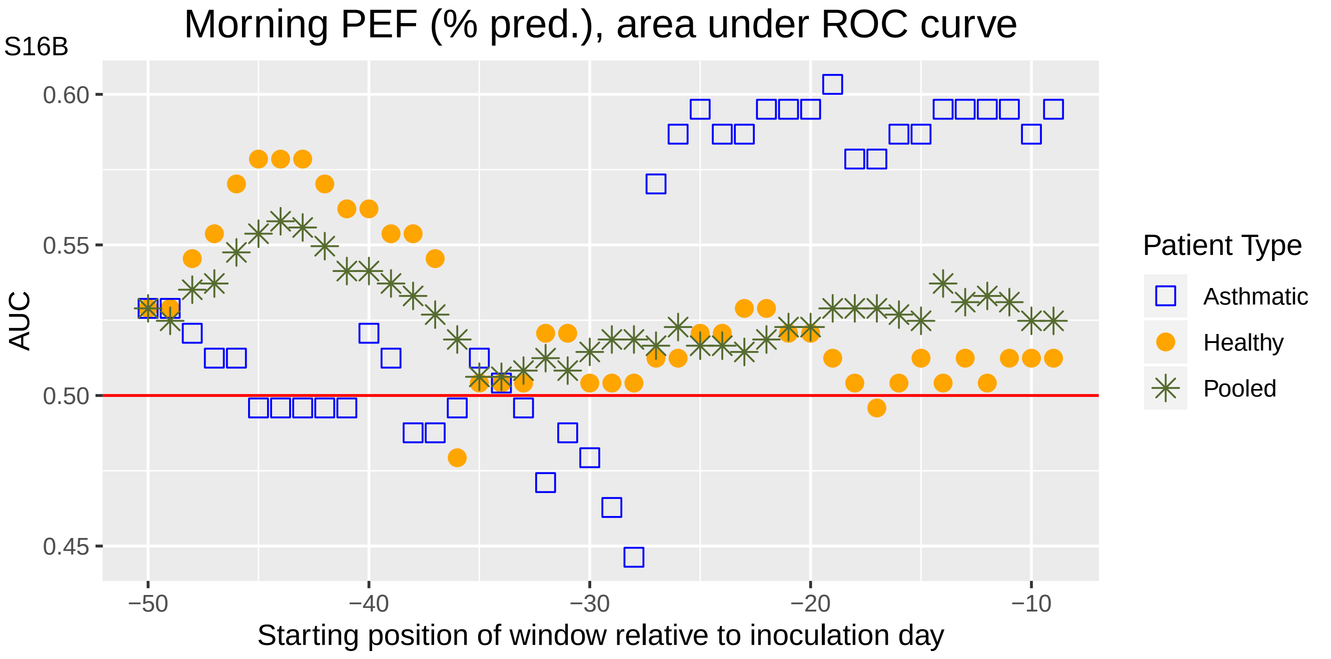
Morning PEF (% pred.), area under ROC curve
S16B
AUC
0.60
0.55
0.50
0.45
−50
−40
−30
−20
−10
Starting position of window relative to inoculation day
Patient Type
Asthmatic
Healthy
Pooled

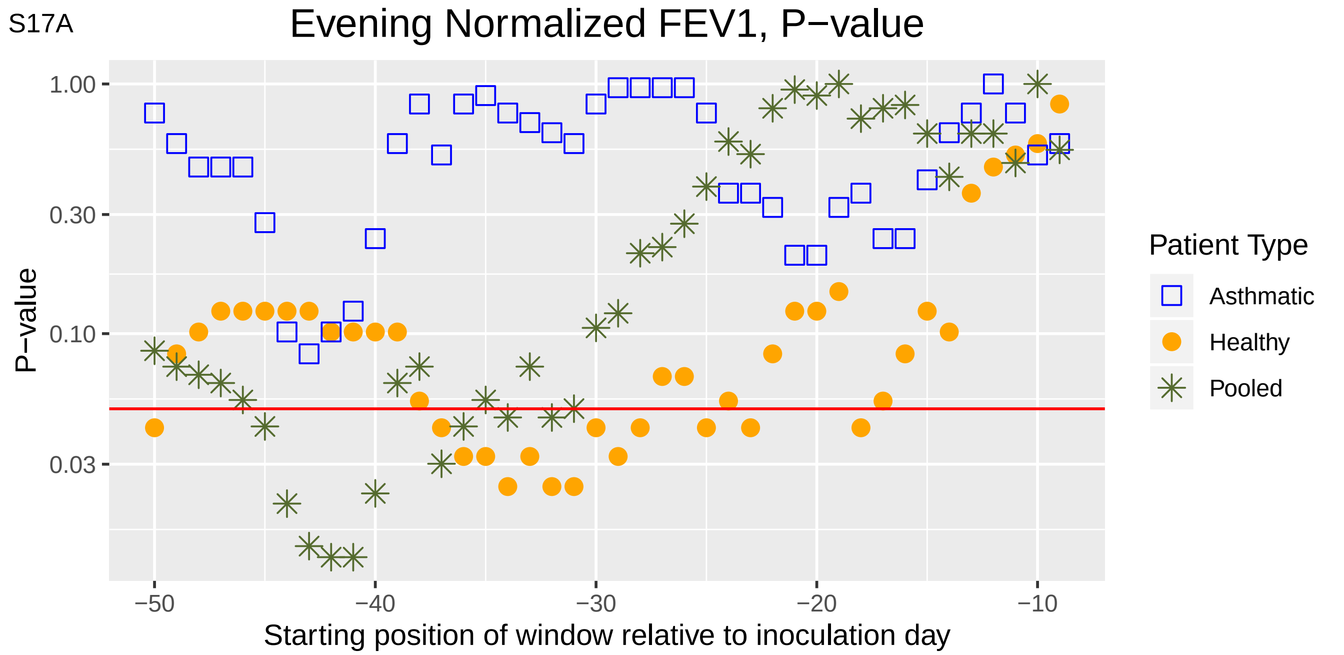
S17A
Evening Normalized FEV1, P−value
P−value
1.00
0.30
0.10
0.03
−50
−40
−30
−20
−10
Starting position of window relative to inoculation day
Patient Type
Asthmatic
Healthy
Pooled

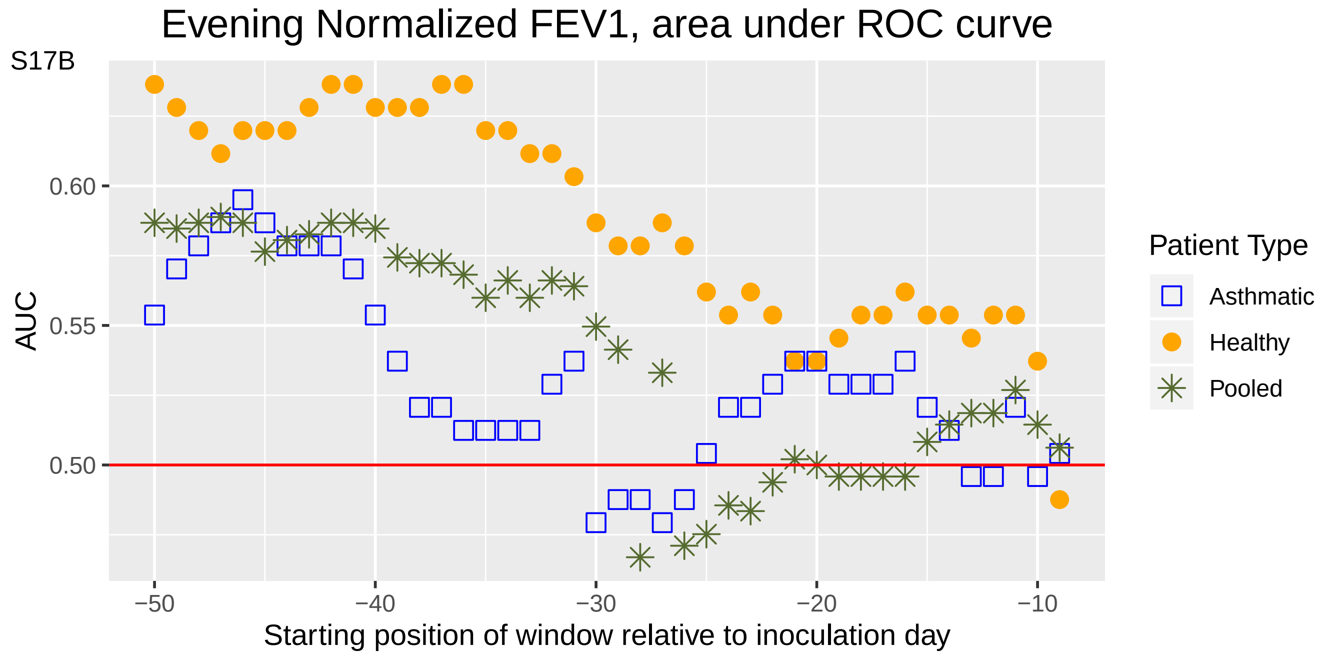
Evening Normalized FEV1, area under ROC curve
S17B
AUC
0.60
0.55
0.50
−50
−40
−30
−20
−10
Starting position of window relative to inoculation day
Patient Type
Asthmatic
Healthy
Pooled

S18A

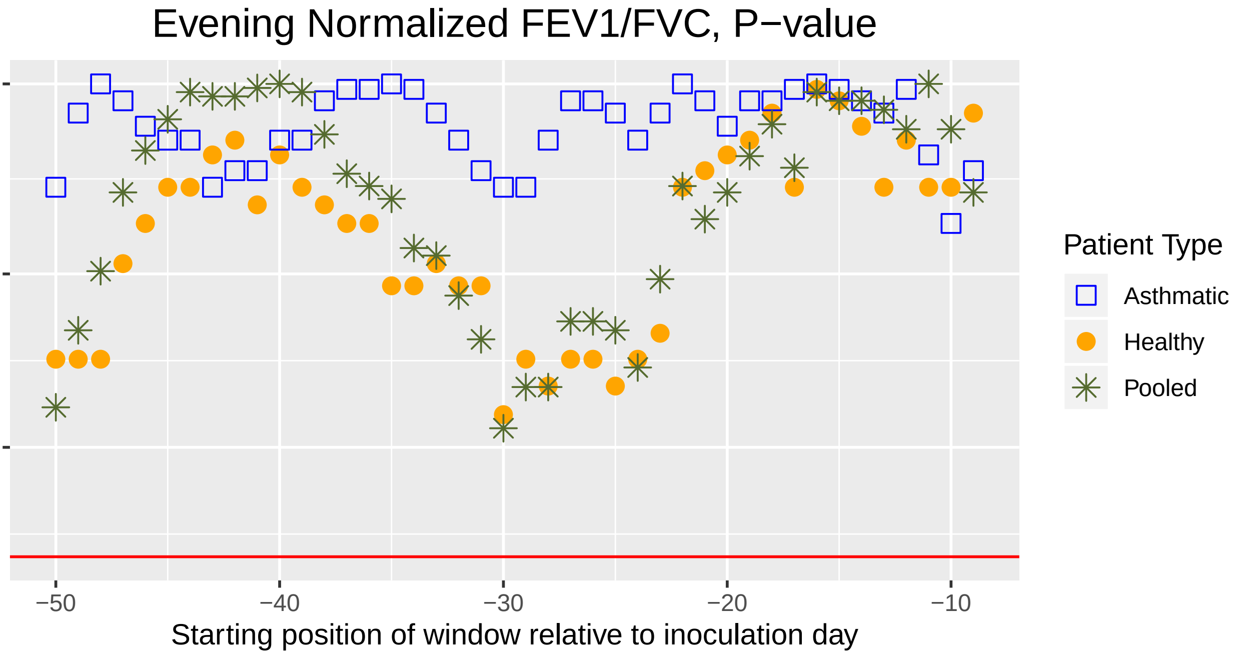
Evening Normalized FEV1/FVC, P−value
P−value
1.0
0.3
0.1
−50
−40
−30
−20
−10
Starting position of window relative to inoculation day
Patient Type
Asthmatic
Healthy
Pooled

S18B

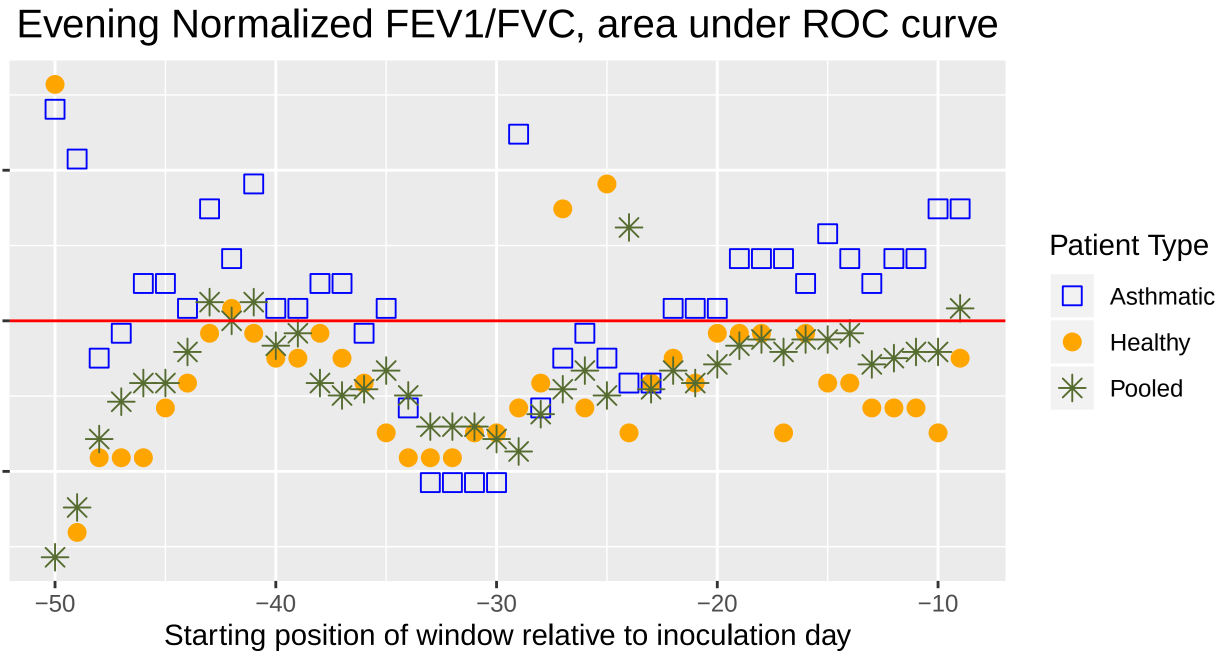
Evening Normalized FEV1/FVC, area under ROC curve
AUC
0.55
0.50
0.45
−50
−40
−30
−20
−10
Starting position of window relative to inoculation day
Patient Type
Asthmatic
Healthy
Pooled

S19A

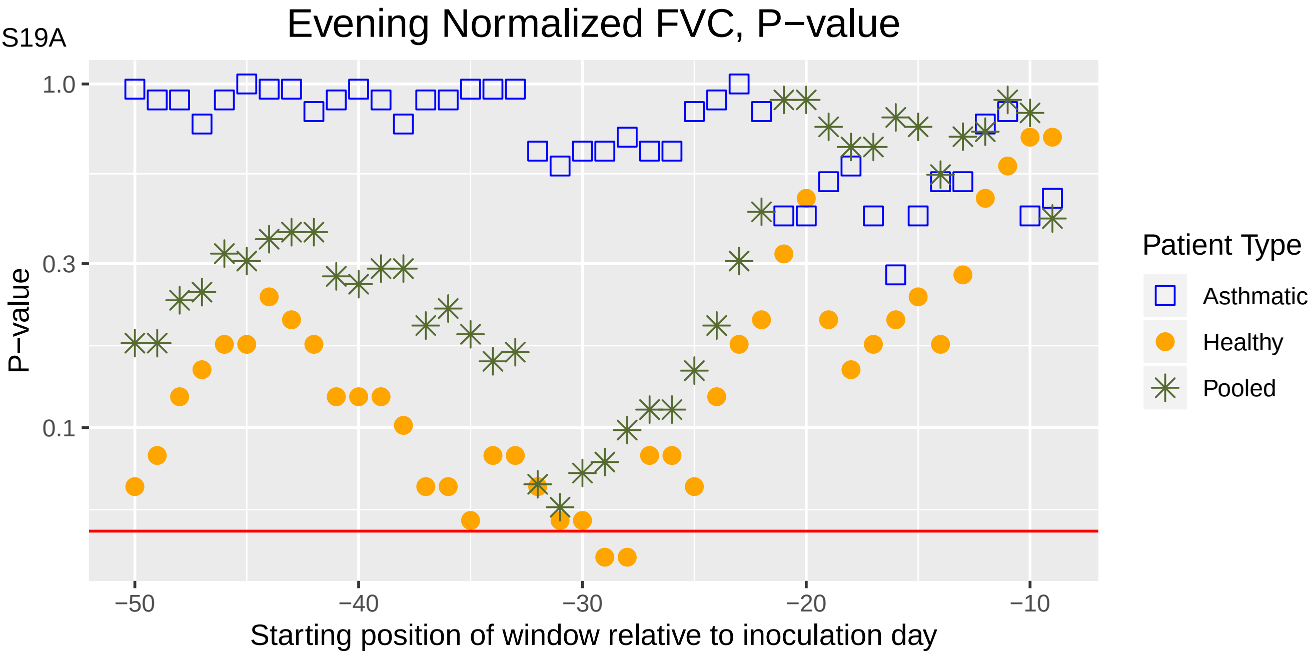
Evening Normalized FVC, P−value
P−value
1.0
0.3
0.1
−50
−40
−30
−20
−10
Starting position of window relative to inoculation day
Patient Type
Asthmatic
Healthy
Pooled

S19B

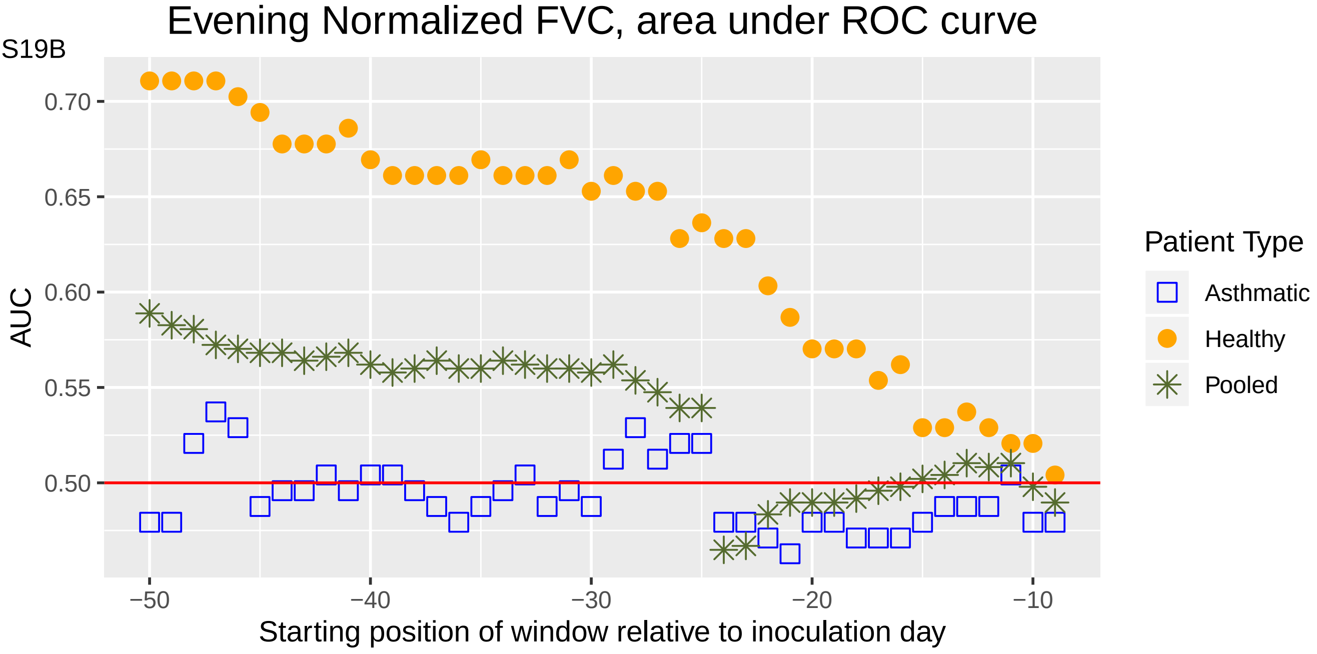
Evening Normalized FVC, area under ROC curve
AUC
0.70
0.65
0.60
0.55
0.50
−50
−40
−30
−20
−10
Starting position of window relative to inoculation day
Patient Type
Asthmatic
Healthy
Pooled

S20A

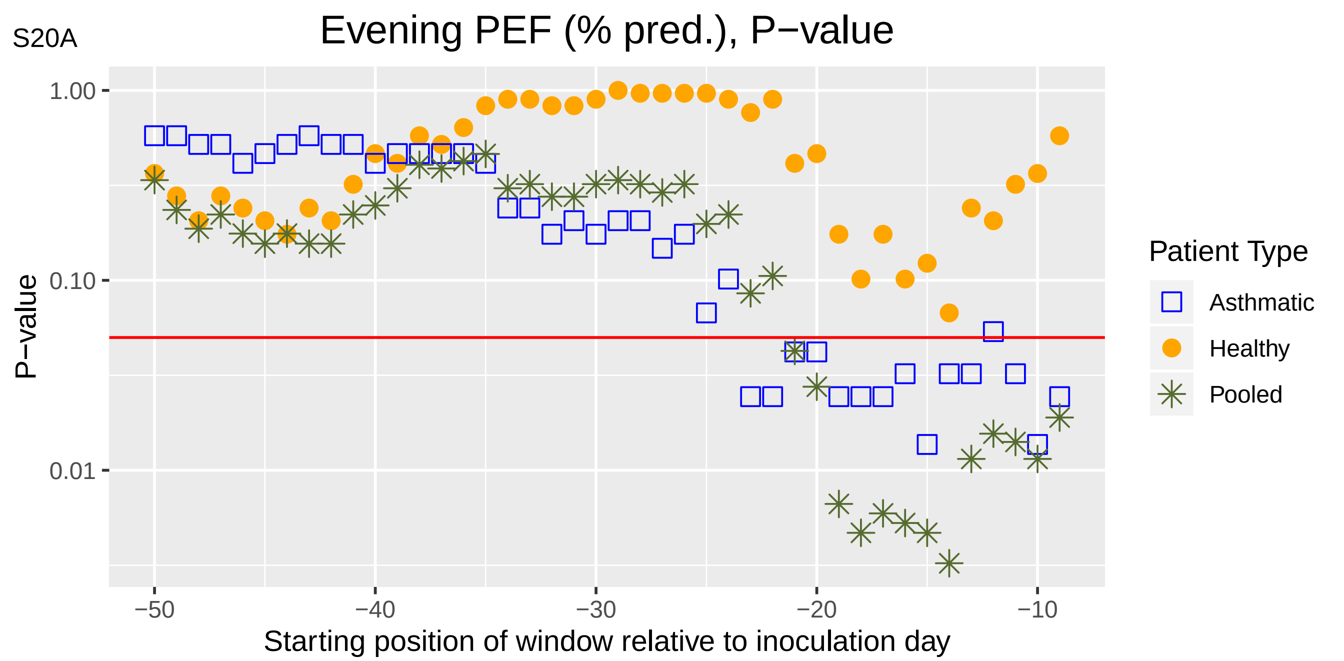
Evening PEF (% pred.), P−value
P−value
1.00
0.10
0.01
−50
−40
−30
−20
−10
Starting position of window relative to inoculation day
Patient Type
Asthmatic
Healthy
Pooled

S20B

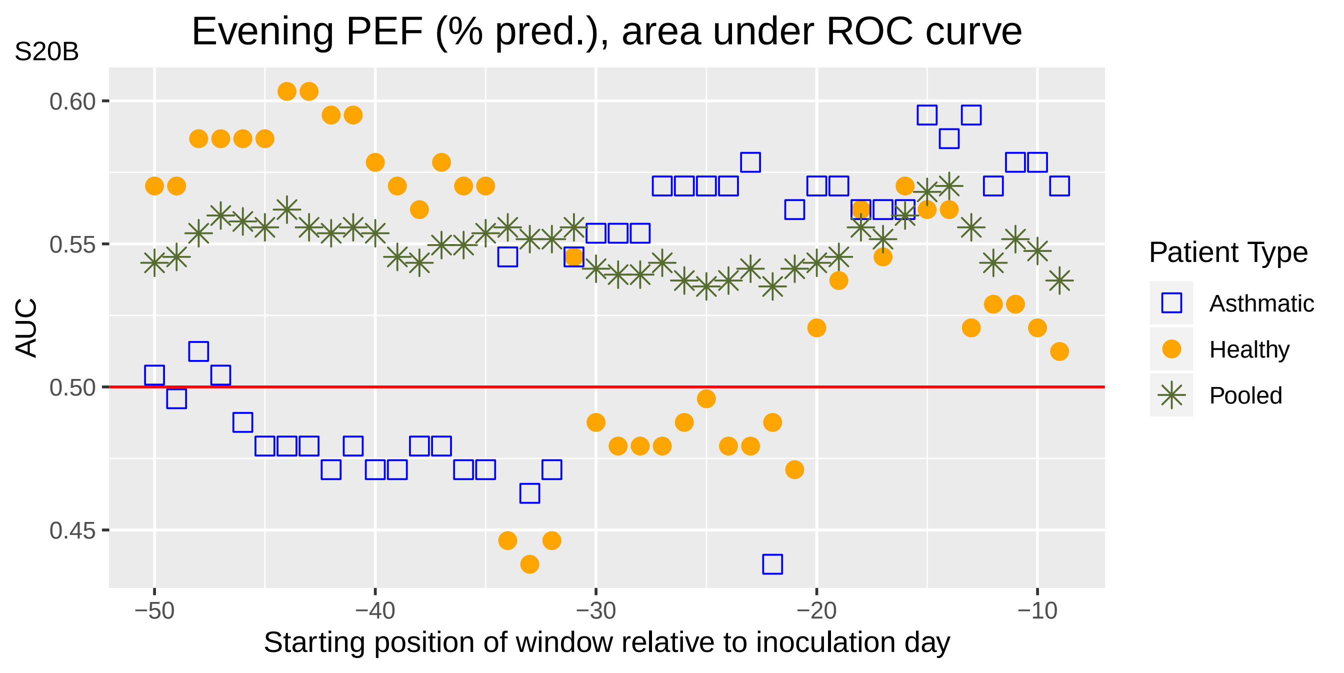
Evening PEF (% pred.), area under ROC curve
AUC
0.60
0.55
0.50
0.45
−50
−40
−30
−20
−10
Starting position of window relative to inoculation day
Patient Type
Asthmatic
Healthy
Pooled
